# Integrating Metabolomics and Gut Microbiota to Reveal the Therapeutic Effect of *Lonicerae japonicae* Flos Against Respiratory Syncytial Virus

**DOI:** 10.3390/metabo16060360

**Published:** 2026-05-27

**Authors:** Yanghai Wang, Yan Gao, Yuting Liang, Bonian Zhao, Lu Liu

**Affiliations:** Institute of Pharmacy, Shandong University of Traditional Chinese Medicine, Jinan 250355, China; 2024100159@sdutcm.edu.cn (Y.W.); 60230003@sdutcm.edu.cn (Y.G.); ytliang@cdutcm.edu.cn (Y.L.)

**Keywords:** *Lonicerae japonicae* Flos, respiratory syncytial virus, metabolomics, gut microbiota

## Abstract

**Objectives**: This study aimed to investigate the therapeutic effects and potential mechanisms of *Lonicerae japonicae* Flos (Jinyinhua, JYH) against respiratory syncytial virus (RSV)-induced pneumonia by integrating lung tissue metabolomics with gut microbiota analysis. **Methods**: An RSV-infected mouse model was established through intranasal inoculation. Lung pathological changes, viral RNA levels, lung index, and inflammatory cytokine levels were evaluated. Untargeted metabolomics and 16S rRNA gene amplicon sequencing were performed to characterize JYH-mediated alterations in pulmonary metabolites and the gut microbiota. Spearman correlation analysis was conducted to assess associations between differentially abundant bacterial genera and significantly altered metabolites. **Results**: JYH alleviated RSV-induced pulmonary histopathological injury, reduced viral RNA levels, decreased lung index and interleukin-6 (IL-6) levels, and increased interferon-γ (IFN-γ) levels. Metabolomic profiling identified 46 differential metabolites, among which 26 showed a reversal trend following JYH administration. These metabolites were mainly enriched in pathways associated with the synaptic vesicle cycle, lysosomal function, and Forkhead box O (FoxO) signaling. Gut microbiota analysis showed that JYH increased microbial richness and diversity, whereas KEGG-based functional prediction indicated that the differentially abundant taxa were primarily involved in amino acid, carbohydrate, and nucleotide metabolism. Moreover, correlation analysis revealed significant associations between key bacterial genera, including *Gemella*, *Sutterella*, and *CC_115*, and differential metabolites such as pyridoxamine, uridine monophosphate (UMP), and argininosuccinic acid. **Conclusions**: JYH may protect against RSV-induced pneumonia by restoring pulmonary metabolic homeostasis and modulating gut microbiota composition. These findings provide new insights into metabolite–microbiota interactions underlying the anti-RSV activity of JYH.

## 1. Introduction

Respiratory syncytial virus (RSV) is a leading cause of acute respiratory tract infections in infants and young children worldwide. In addition to typical respiratory manifestations, such as cough, wheezing, and dyspnea, RSV infection may also be accompanied by gastrointestinal symptoms, including nausea, vomiting, diarrhea, and constipation. Despite its substantial disease burden, effective RSV-specific antiviral therapies remain limited, and the prevention and treatment of RSV infection continue to pose considerable challenges.

Accumulating evidence indicates that RSV infection affects not only the respiratory tract but also the intestinal microenvironment. RSV-induced respiratory inflammation has been linked to intestinal pathological alterations and gut microbiota dysbiosis [1]. In particular, RSV infection can reduce gut microbial diversity and influence the development and progression of respiratory disease through multiple mechanisms, including immune modulation and altered short-chain fatty acid production [2]. Conversely, probiotic interventions have been shown to enhance intestinal barrier integrity, modulate host immune responses, restore gut–lung microecological balance, and attenuate pulmonary inflammation, thereby exerting protective effects against RSV infection [3]. Collectively, these findings suggest that the gut–lung axis may play an important role in the pathogenesis and therapeutic modulation of RSV-associated respiratory disease.

Metabolomics, first proposed by Nicholson and colleagues, focuses on the comprehensive characterization of small-molecule metabolites and their dynamic alterations in biological systems under physiological and pathological conditions. In RSV-related research, metabolomics has been increasingly applied to identify infection-associated metabolic perturbations across diverse biological samples. For example, specific lipid alterations have been detected in the lung tissues and plasma of RSV-infected mice, particularly involving metabolites related to pulmonary surfactants, suggesting that disruption of the pulmonary surfactant system may contribute to RSV-induced respiratory inflammation [4]. In addition, alterations in plasma intermediates involved in sialic acid biosynthesis, together with disturbances in urinary citrate and nucleotide metabolites, have been closely associated with RSV infection severity [5]. These findings highlight the potential of metabolomics for revealing key metabolic pathways involved in RSV pathogenesis and therapeutic intervention.

*Lonicerae japonicae* Flos, commonly known as Jinyinhua (JYH), is a traditional medicinal and edible plant with a long history of clinical and dietary use. Its bioactive constituents have been reported to exhibit diverse pharmacological activities, including anti-inflammatory, antiviral, antibacterial, antioxidant, antitumor, cardioprotective, and hepatoprotective effects [6]. JYH is widely used in the pharmaceutical industry as a major component of several Chinese patent medicines, such as Shuanghuanglian Oral Liquid, Yinhuang Injection, and JYH Dew. In addition to its medicinal value, JYH is cultivated as an important economic crop in Asia and Europe and is widely used in the food, personal care, and cosmetics industries, particularly in floral teas and herbal beverages [7]. Previous studies have demonstrated that JYH extracts [8], polysaccharides [9], and chlorogenic acid [10] exhibit notable anti-RSV activity. However, the mechanisms underlying the protective effects of JYH against RSV infection remain incompletely understood.

Therefore, in the present study, an RSV-infected mouse model was established to investigate the therapeutic effects and potential mechanisms of JYH against RSV infection. LC–MS-based metabolomics was employed to characterize endogenous metabolic alterations in lung tissues and to identify differential metabolites and related metabolic pathways associated with RSV infection and JYH intervention. Meanwhile, 16S rRNA gene sequencing was performed on mouse intestinal contents to assess alterations in the gut microbial community. Furthermore, integrated correlation analysis between differential metabolites and gut microbiota was conducted to explore potential interactions between pulmonary metabolic remodeling and intestinal microbial regulation. This study provides new insights into the anti-RSV effects of JYH from the perspectives of lung tissue metabolomics and gut microbiota regulation.

## 2. Materials and Methods

### 2.1. Materials

Mouse interleukin-6 (IL-6) and interferon-γ (IFN-γ) ELISA kits were purchased from Hangzhou Lianke Biotechnology Co., Ltd. (Hangzhou, China). Methanol and acetonitrile were obtained from Thermo Fisher Scientific (Waltham, MA, USA), and ammonium formate was purchased from Sigma-Aldrich (St. Louis, MO, USA). Formic acid was of LC–MS grade, and all other reagents were of analytical grade unless otherwise specified.

*Lonicerae japonicae* Flos (Jinyinhua, JYH) was purchased from a traditional Chinese medicine market in Anhui, China. Ribavirin granules were obtained from Sichuan Baili Pharmaceutical Co., Ltd. (Sichuan, China). Cholic acid-2,2,3,4,4-d5 and succinic acid-2,2,3,3-d4 were purchased from Sigma-Aldrich. DL-tryptophan-2,3,3-d3 and DL-methionine-3,3,4,4-d4 were obtained from CDN Isotopes (Pointe-Claire, QC, Canada). L-phenylalanine-ring-d5 and choline chloride were purchased from Cambridge Isotope Laboratories (Tewksbury, MA, USA).

Agarose, TAE buffer, and the Quant-iT PicoGreen dsDNA Assay Kit were purchased from Invitrogen (Carlsbad, CA, USA). DNA Marker was obtained from Takara Bio Inc. (Shiga, Japan), and Q5^®^ High-Fidelity DNA Polymerase was purchased from New England Biolabs or NewBio-Era Biotechnology Co., Ltd. (Beijing, China). Real-time quantitative PCR was performed using a CFX Connect real-time quantitative fluorescence PCR system (Bio-Rad Laboratories, Hercules, CA, USA).

### 2.2. Animals

Male BALB/c mice weighing 10 ± 2 g and aged 2 weeks were purchased from Beijing Vital River Laboratory Animal Technology Co., Ltd. (Beijing, China). All animals were housed under specific pathogen-free conditions at the Experimental Animal Center of Shandong University of Traditional Chinese Medicine. Animal care and all experimental procedures were conducted in accordance with institutional guidelines. The study protocol was approved by the Animal Ethics Committee of Shandong University of Traditional Chinese Medicine.

### 2.3. Preparation of JYH Extract and Ribavirin Solution

According to the Pharmacopoeia of the People’s Republic of China, the maximum recommended daily dose of JYH for adults is 15 g. The mouse-equivalent dose was calculated using the following formula: *d_B_* = *d_A_* × (*R_B_*/*R_A_*) × (*W_A_*/*W_B_*)^1/3^, where *d_A_* and *d_B_* represent the doses for humans and mice, respectively; *R_A_* and *R_B_* represent the body shape coefficients of humans and mice, respectively; and *W_A_* and *W_B_* represent the body weights of humans and mice, respectively. Based on this calculation, the mouse-equivalent dose was determined to be 2.68 g/kg. Previous studies have shown that a dose equivalent to four times the calculated mouse-equivalent dose produces optimal therapeutic effects in mice [11]. Therefore, JYH was administered at 10.72 g/kg in the present study.

For extraction, 15 g of JYH powder was accurately weighed and transferred to a round-bottom flask. Boiling water was added at a solid-to-liquid ratio of 1:200 (*w*/*v*), and the mixture was refluxed for 30 min. The extract was filtered, and the filtrate was concentrated to 1 g/mL using a rotary evaporator. Ribavirin was dissolved in distilled water and administered at 0.08 g/kg as the positive control drug.

### 2.4. Animal Grouping and Establishment of the RSV-Infected Mouse Model

**Figure 1:**
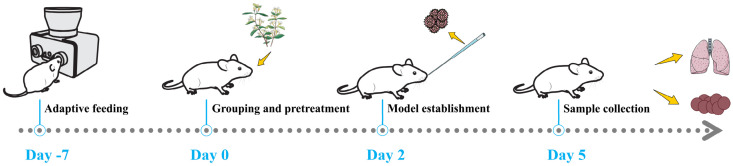
Flow chart of the experimental design.

Thirty-two two-week-old male BALB/c mice weighing 10–12 g were randomly assigned to four groups using a random number table method: control group, model group, ribavirin group, and JYH group, with eight mice per group. Mice in the ribavirin and JYH groups were pretreated by oral gavage for two consecutive days with ribavirin solution at 0.08 g/kg and JYH extract at 10.72 g/kg, respectively. Mice in the control and model groups received an equivalent volume of distilled water. On day 3, mice in the model, ribavirin, and JYH groups were intranasally inoculated with 35 μL of RSV suspension (10^−6^ TCID_50_), whereas mice in the control group received an equivalent volume of saline. After viral inoculation, mice in the JYH and ribavirin groups continued to receive JYH extract or ribavirin solution once daily by oral gavage for three consecutive days, while mice in the control and model groups received an equivalent volume of distilled water. During the experimental period, the general condition of the mice, including mental status, coat appearance, and food intake, was monitored daily. The experimental design is illustrated in Figure 1.

### 2.5. Sample Collection

Thirty minutes after the final administration, the mice were anesthetized, and biological samples were collected. Lung tissues were excised and weighed immediately. A portion of the left lung was fixed in 4% paraformaldehyde for histopathological analysis. The remaining lung tissues were snap-frozen in liquid nitrogen and stored at −80 °C for subsequent ELISA and metabolomic analyses. Cecal contents were collected from each mouse, snap-frozen in liquid nitrogen, and stored at −80 °C for 16S rRNA gene sequencing.

### 2.6. Histopathological Analysis

Lung tissues were fixed in 4% paraformaldehyde, embedded in paraffin, sectioned, and stained with hematoxylin and eosin (H&E). Histopathological changes were observed under a light microscope and evaluated according to predefined histological scoring criteria, ranging from 0 (absent) to 5 (severe).

### 2.7. Relative Viral RNA Expression in Lung Tissues

Total RNA was extracted from lung tissues according to the manufacturer’s protocol. The extracted RNA was reverse-transcribed into cDNA using the PrimeScript RT Reagent Kit (Takara Bio Inc., Shiga, Japan). Quantitative real-time PCR (qRT-PCR) was performed using SYBR Premix Ex Taq™ II (Takara Bio Inc., Shiga, Japan) according to the manufacturer’s protocol. The qRT-PCR cycling conditions were as follows: initial denaturation at 95 °C for 30 s, followed by 40 cycles of denaturation at 95 °C for 5 s and annealing/extension at 60 °C for 30 s. The primer sequences used in this study are listed in Table 1.

### 2.8. Enzyme-Linked Immunosorbent Assay

Lung tissues from each group were placed in centrifuge tubes, and ice-cold phosphate-buffered saline (PBS) containing 1% phenylmethylsulfonyl fluoride (PMSF) was added at five times the tissue weight. The tissues were homogenized at 4 °C using a tissue grinder and then centrifuged at 4 °C. The supernatants were collected, and the concentrations of IL-6 and IFN-γ were measured using commercial ELISA kits according to the manufacturer’s protocols.

### 2.9. Metabolomic Analysis

Lung tissue samples were placed in 2 mL centrifuge tubes containing steel beads and homogenized using a tissue grinder at 55 Hz for 60 s. Approximately 88.3–101.7 mg of each homogenized sample was accurately weighed and transferred into a 2 mL centrifuge tube. Subsequently, 0.6 mL of methanol containing internal standards was added. The mixture was vortexed for 30 s and centrifuged at 12,000 rpm and 4 °C for 10 min. The supernatant was filtered through a 0.22 μm membrane and transferred to an autosampler vial for UHPLC–MS analysis. Quality control (QC) samples were prepared by pooling 20 μL aliquots from each of the 24 analytical samples and were stored at 4 °C before analysis. Metabolomic profiling was performed using an ultra-high-performance liquid chromatography–Orbitrap high-resolution mass spectrometry system.

Chromatographic separation was performed using a Thermo Ultimate 3000 UHPLC system equipped with an ACQUITY UPLC^®^ HSS T3 column (1.8 μm, 2.1 × 150 mm, Waters Corporation, Milford, MA, USA). The autosampler temperature was maintained at 8 °C, the flow rate was set at 0.25 mL/min, the column temperature was maintained at 40 °C, and the injection volume was 2 μL.

In positive ion mode, the mobile phases consisted of 0.1% formic acid in water as phase C and 0.1% formic acid in acetonitrile as phase D. In negative ion mode, the mobile phases consisted of 5 mM ammonium formate in water as phase A and acetonitrile as phase B. The gradient elution program was as follows: 0–1 min, 2% B/D; 1–9 min, 2–50% B/D; 9–12 min, 50–98% B/D; 12–13.5 min, 98% B/D; 13.5–14 min, 98–2% B/D. Re-equilibration was performed at 2% D from 14 to 20 min in positive ion mode and at 2% B from 14 to 17 min in negative ion mode.

Mass spectrometric analysis was conducted using a Thermo Q Exactive Focus mass spectrometer (Thermo Fisher Scientific, Waltham, MA, USA) equipped with an electrospray ionization (ESI) source operated in both positive and negative ion modes. The spray voltage was set to 3.50 kV in positive ion mode and 2.50 kV in negative ion mode. The sheath gas and auxiliary gas pressures were set at 30 arb and 10 arb, respectively. The capillary temperature was maintained at 325 °C, and the resolution was set to 35,000. Full-scan mass spectra were acquired over an *m*/*z* range of 81–1000. Higher-energy collisional dissociation (HCD) was applied for MS/MS fragmentation at a collision energy of 30 eV, and dynamic exclusion was used to reduce redundant MS/MS acquisition.

Raw MS data were imported into ProteoWizard software (version 3.0.8789, ProteoWizard, Seattle, WA, USA) and converted to mzXML format. Peak detection, filtering, extraction, and alignment were performed using the XCMS package in R software (version 3.3.2) with the following parameters: bw = 2, ppm = 15, peakwidth = c(5, 30), mzwid = 0.015, mzdiff = 0.01, and method = centWave. Scaling normalization was performed to enable comparison among variables with different abundance ranges. Multivariate statistical analysis was conducted using SIMCA-P software (version 13.0, Umetrics, Malmö, Sweden).

Potential differential metabolites were screened according to the criteria of variable importance in projection (VIP) > 1, *p* < 0.05, and false discovery rate (FDR) ≤ 0.05. Metabolite identification was performed based on accurate mass information with a mass error of <15 ppm, MS/MS fragment ions, and comparisons with public databases, including METLIN and MoNA. Pathway enrichment analysis of the identified differential metabolites was performed using the Kyoto Encyclopedia of Genes and Genomes (KEGG) database and the MetaboAnalyst pathway.

### 2.10. Gut Microbiota Analysis

Genomic DNA was extracted from cecal content samples using a commercial DNA extraction kit according to the manufacturer’s protocol. The V3–V4 hypervariable region of the bacterial 16S rRNA gene was amplified using the primers 338F 5′-ACTCCTACGGGAGGCAGCA-3′, and 806R 5′-GGACTACHVGGGTWTCTAAT-3′, together with sample-specific barcode sequences. Sequencing libraries were constructed using the Illumina TruSeq Nano DNA Library Prep Kit (Illumina Inc., San Diego, CA, USA), and paired-end sequencing was performed on an Illumina MiSeq or NovaSeq platform.

Raw sequencing data were processed using the DADA2 pipeline in QIIME 2. Sequences were denoised, quality-filtered, merged, and chimera-filtered. To ensure comparability across samples at an even sequencing depth, the ASV/OTU abundance table was rarefied using the qiime feature-table rarefy function in QIIME 2, with the rarefaction depth set to 95% of the minimum library size. Alpha and beta diversity analyses were performed to evaluate microbial diversity and community structure across samples. Differences in bacterial taxa among groups were assessed using Venn diagram analysis, microbial composition analysis, and linear discriminant analysis effect size (LEfSe). Based on the taxonomic profiles, functional prediction of the gut microbiota was subsequently performed.

### 2.11. Statistical Analysis

Statistical analyses were performed using GraphPad Prism software, version 5.0. The normality of multi-group experimental data was assessed using the Shapiro–Wilk and Kolmogorov–Smirnov tests. For data that did not follow a normal distribution, the non-parametric Kruskal–Wallis test was used, followed by Dunn’s multiple comparison test for post hoc analysis. For normally distributed data, homogeneity of variance was further assessed. When variances were homogeneous, one-way analysis of variance (ANOVA) followed by the Newman–Keuls post hoc test was used for comparisons among groups. When variances were unequal, Welch’s corrected unpaired *t*-test was applied. A *p*-value of <0.05 was considered statistically significant.

## 3. Results

### 3.1. JYH Inhibited RSV Infection Progression and Alleviated Pulmonary Pathological Injury in Mice

To evaluate the therapeutic effects of JYH against RSV infection, the general condition of mice was monitored daily. Lung tissues were collected 0.5 h after the final administration for histopathological examination and inflammatory cytokine analysis.

Mice in the control group exhibited normal behavior, regular food intake, agile movement, and smooth fur. During model establishment, RSV-infected mice showed reduced appetite, lethargy, decreased responsiveness, and rapid breathing. In contrast, mice treated with ribavirin or JYH displayed milder infection-related symptoms than those in the model group.

**Figure 2:**
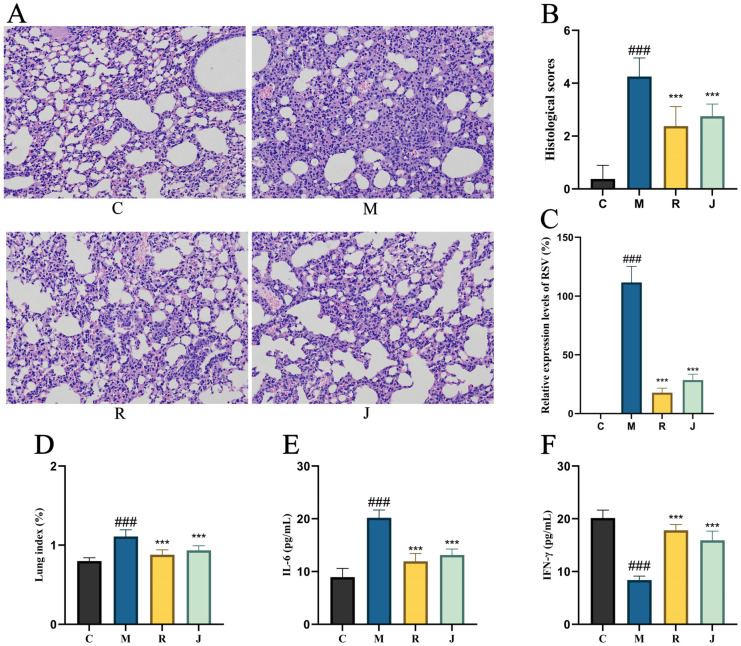
Therapeutic effects of JYH against RSV infection in mice. (**A**) Representative hematoxylin and eosin (H&E)-stained lung tissue sections; original magnification, ×200. (**B**) Histological injury scores. (**C**) Relative RSV RNA levels in lung tissues. (**D**) Lung index. (**E**) IL-6 levels. (**F**) IFN-γ levels. C, control group; M, model group; R, ribavirin group; J, JYH group. Data are presented as the mean ± SD, *n* = 8. ^###^ indicates *p* < 0.001 vs. control group; *** indicates *p* < 0.001 vs. model group.

Histopathological examination showed that JYH intervention alleviated RSV-induced lung injury. In the control group, the alveolar architecture remained intact, with no obvious alveolar wall thickening or inflammatory cell infiltration. In contrast, the model group exhibited marked alveolar wall thickening, extensive inflammatory cell infiltration, partial alveolar atrophy, compensatory alveolar enlargement, irregular alveolar morphology, and eosinophilic flocculent exudates. Necrotic cells with nuclear fragmentation and dissolution were also observed, whereas mild inflammatory infiltration was detected in the bronchi. After ribavirin or JYH treatment, inflammatory cell infiltration and alveolar wall thickening were substantially reduced, and the overall histological structure of lung tissues was improved (Figure 2A). Consistently, histological scores were lower in the ribavirin and JYH groups than in the model group (Figure 2B).

Compared with the control group, RSV infection significantly increased the relative viral RNA level in lung tissues, the lung index, and IL-6 levels, while markedly reducing IFN-γ levels (*p* < 0.001). Specifically, the lung index increased from 0.80 ± 0.04 in the control group to 1.11 ± 0.09 in the model group, while IL-6 levels increased from 8.94 ± 1.64 to 20.19 ± 1.46. Conversely, IFN-γ levels decreased from 20.13 ± 1.50 in the control group to 8.40 ± 1.75 in the model group. These results confirmed the successful establishment of RSV-induced pulmonary inflammation.

Compared with the model group, both ribavirin and JYH significantly reduced the relative viral RNA levels in lung tissues to 17.80 ± 3.80 and 28.55 ± 4.96, respectively. In addition, ribavirin and JYH decreased the lung index to 0.88 ± 0.06 and 0.93 ± 0.06, respectively, and reduced IL-6 levels to 11.94 ± 1.47 and 13.14 ± 1.12, respectively (*p* < 0.001). Meanwhile, IFN-γ levels were significantly increased to 17.82 ± 1.10 and 15.91 ± 1.75 in the ribavirin and JYH groups, respectively (*p* < 0.001). These findings suggest that JYH suppressed RSV replication and attenuated RSV-induced pulmonary inflammation in mice (Figure 2C–F).

### 3.2. Effects of JYH on Lung Tissue Metabolomics in RSV-Infected Mice

**Figure 3:**
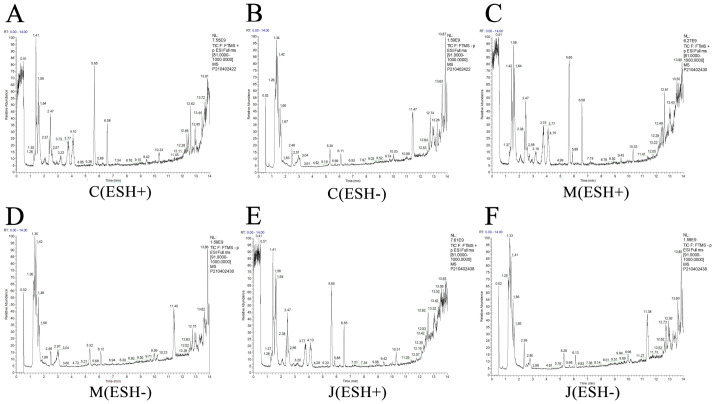
Total ion chromatogram of lung tissue samples. (**A**) Total ion chromatogram of lung tissue samples from the control group in positive ion mode. (**B**) Total ion chromatogram of lung tissue from the control group in negative ion mode. (**C**) Total ion chromatogram of lung tissue samples from the model group in positive ion mode. (**D**) Total ion chromatogram of lung tissue from the model group in negative ion mode. (**E**) Total ion chromatogram of lung tissue samples from the JYH group in positive ion mode. (**F**) Total ion chromatogram of lung tissue from the JYH group in negative ion mode. C, control group; M, model group; J, JYH group.

To identify metabolites differentially altered in response to RSV infection and to evaluate the modulatory effects of JYH treatment, LC–MS-based metabolomic analysis was performed on lung tissue samples from the control, model, and JYH groups (Figure 3).

Quality control (QC) analysis was first conducted to ensure the reliability and reproducibility of the metabolomic data. During data acquisition, pooled QC samples were periodically analyzed to evaluate instrumental stability and methodological consistency. In the principal component analysis (PCA) score plot, the QC samples were tightly clustered, indicating good instrumental stability, analytical reproducibility, and overall data reliability. Quality assurance (QA) was further performed based on the QC results. Features with a relative standard deviation (RSD) greater than 30% in the QC samples were excluded because of poor reproducibility. After filtering, approximately 60% of the detected features showed an RSD below 30%, indicating that the dataset was suitable for subsequent analysis (Supplementary Figure S1).

**Figure 4:**
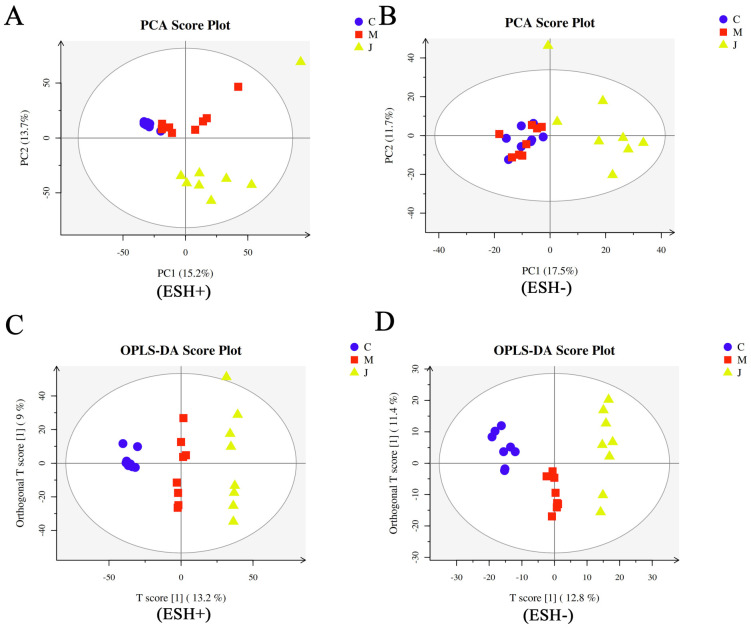
Multivariate statistical analysis of lung tissue metabolomic profiles. (**A**,**B**) Principal component analysis (PCA) score plots of lung tissue samples in positive and negative ion modes, respectively. (**C**,**D**) Orthogonal partial least squares discriminant analysis (OPLS-DA) score plots of lung tissue samples in positive and negative ion modes, respectively. The model parameters were R^2^ = 0.992 and Q^2^ = 0.863 in positive ion mode, and R^2^ = 0.985 and Q^2^ = 0.780 in negative ion mode. C, control group; M, model group; J, JYH group. *n* = 8.

PCA revealed distinct metabolic profiles among the control, model, and JYH groups, suggesting that RSV infection induced marked metabolic alterations in lung tissues and that JYH intervention partially modulated these alterations (Figure 4A,B). Orthogonal partial least squares discriminant analysis (OPLS-DA) further showed separation between the model and JYH groups in both positive and negative ion modes. The OPLS-DA models showed R^2^ = 0.992 and Q^2^ = 0.863 in positive ion mode, and R^2^ = 0.985 and Q^2^ = 0.780 in negative ion mode (Figure 4C,D).

**Figure 5:**
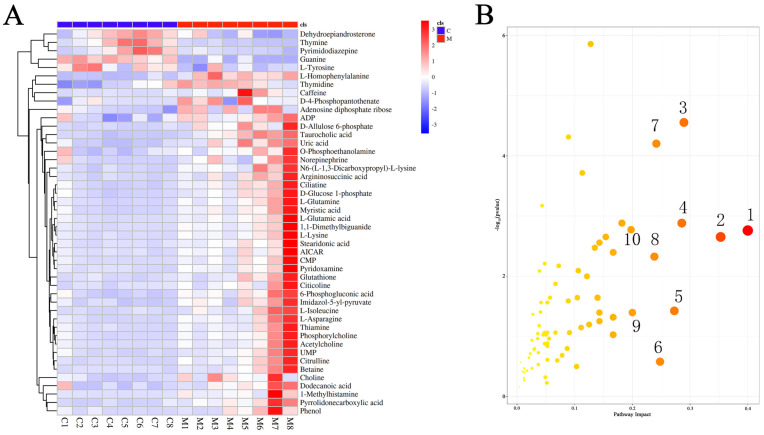
Differential metabolite and metabolic pathway analyses of lung tissue samples. (**A**) Heatmap of differential metabolites between the control and model groups. (**B**) Bubble plot of enriched metabolic pathways between the model and JYH groups. The numbered pathways are as follows: 1. Glutamatergic synapse 2. GABAergic synapse 3. Arginine biosynthesis 4. Nicotine addiction 5. Glutathione metabolism 6. Starch and sucrose metabolism 7. Alanine, aspartate and glutamate metabolism 8. D-Glutamine and D-glutamate metabolism 9. FoxO signaling Pathway 10. Pyrimidine metabolism. C, control group; M, model group. Differential metabolites were screened using VIP > 1, *p* < 0.05, and FDR ≤ 0.05.

Differential metabolites were screened according to the criteria of VIP > 1, *p* < 0.05, and FDR ≤ 0.05. A total of 46 significantly altered metabolites were identified between the control and model groups, including 41 upregulated and 5 downregulated metabolites, indicating substantial metabolic disturbances following RSV infection (Figure 5A). In addition, 26 differential metabolites were identified between the JYH and model groups, including phenol, pyridoxamine, phosphorylcholine, and thymidine, suggesting that these metabolites may contribute to JYH-mediated metabolic regulation (Supplementary Figure S2).

KEGG pathway enrichment analysis using the MetPA platform showed that the differential metabolites were mainly enriched in pathways related to glutamatergic synapse, gamma-aminobutyric acid (GABA)ergic synapse, arginine biosynthesis, nicotine addiction, glutathione metabolism, starch and sucrose metabolism, alanine, aspartate, and glutamate metabolism, D-glutamine and D-glutamate metabolism, FoxO signaling pathway, and pyrimidine metabolism (Figure 5B). These findings suggest that JYH may exert protective effects against RSV infection by modulating amino acid metabolism, nucleotide metabolism, oxidative stress-related pathways, and signaling pathways associated with cellular homeostasis.

### 3.3. Effects of JYH on the Gut Microbiota in RSV-Infected Mice

Following oral administration, JYH extract may directly interact with the gastrointestinal environment before systemic absorption, thereby influencing gut microbial composition and function. To investigate the effects of RSV infection and JYH treatment on the intestinal microbiota, 16S rRNA gene sequencing was performed on cecal content samples from mice.

#### 3.3.1. Microbial Diversity Analysis

**Figure 6:**
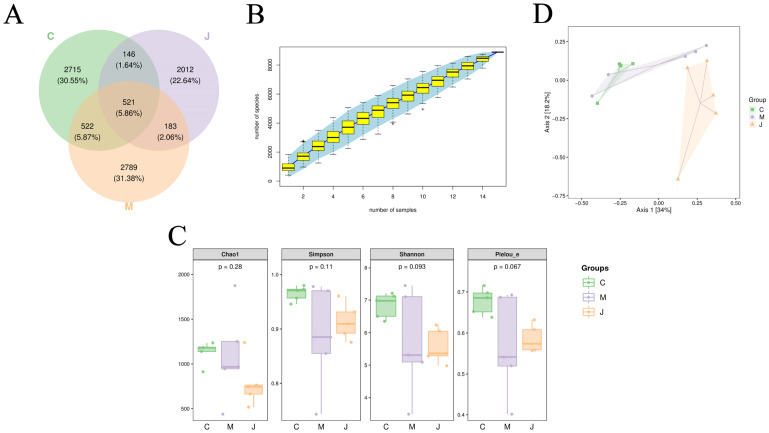
The Effects of JYH on the Gut Microbiome of Mice Infected with RSV. (**A**) Venn diagram showing shared and unique OTUs among groups. (**B**) Species accumulation curves. (**C**) Alpha diversity analysis, including the Chao1, Shannon, Simpson, and Pielou’s evenness indices. (**D**) Beta diversity analysis based on principal coordinate analysis (PCoA), showing differences in microbial community structure among groups. C, control group; M, model group; J, JYH group. *n* = 5. *p*-values for group comparisons are indicated in the figure.

Venn diagram analysis was used to compare the number of operational taxonomic units (OTUs) among groups. A total of 2715, 2789, and 2012 OTUs were detected in the control, model, and JYH groups, respectively, with 521 OTUs shared among all three groups (Figure 6A). Species accumulation curves approached saturation, indicating that the sequencing depth and sample size were sufficient for subsequent microbial community analyses (Figure 6B).

Alpha diversity was assessed using the Chao1, Shannon, Simpson, and Pielou’s evenness indices to evaluate microbial richness, diversity, and evenness within samples. The Chao1 index was decreased in both the model and JYH groups, suggesting that RSV infection and JYH intervention affected gut microbial richness. Compared with the control group, the Shannon, Simpson, and Pielou’s evenness indices were reduced in the model group, indicating decreased microbial diversity and evenness following RSV infection. These changes were partially restored following JYH treatment, suggesting that JYH improved gut microbial diversity and evenness in RSV-infected mice (Figure 6C).

Principal coordinate analysis (PCoA) revealed distinct clustering of gut microbial communities among the control, model, and JYH groups. The clear separation between the model and JYH groups suggested that JYH treatment reshaped gut microbiota composition in RSV-infected mice (Figure 6D). Consistently, Adonis analysis showed significant differences in microbial community structure among groups, with an R^2^ value of 0.1795, an F value of 1.3132, and a Pr(>F) value of 0.003, further supporting treatment-associated shifts in gut microbial composition.

#### 3.3.2. Gut Microbiota Composition Analysis

**Figure 7:**
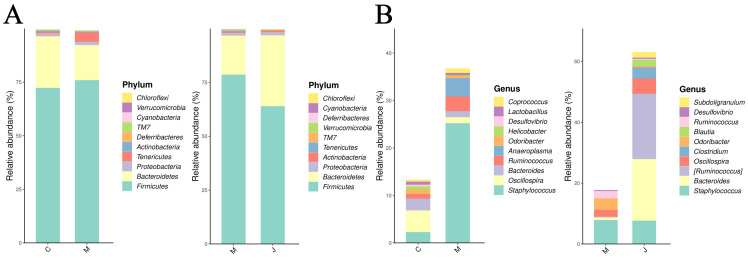
Analysis of gut microbiota in mice infected with RSV. (**A**) Relative abundance of gut microbiota at the phylum level. The corresponding currently valid phylum names are shown in parentheses: *Chloroflexi* (*Chloroflexota*), *Verrucomicrobia* (*Verrucomicrobiota*), *Cyanobacteria* (*Cyanobacteriota*), *Deferribacteres* (*Deferribacterota*), *Actinobacteria* (*Actinomycetota*), *Tenericutes* (*Mycoplasmatota*), *Proteobacteria* (*Pseudomonadota*), *Bacteroidetes* (*Bacteroidota*), and *Firmicutes* (*Bacillota*). (**B**) Relative abundance of gut microbiota at the genus level. C, control group; M, model group; J, JYH group.

The relative abundance of the gut microbiota was further analyzed at the phylum and genus levels. At the phylum level, RSV infection markedly altered gut microbial composition. Compared with the control group, the model group showed increased relative abundances of *Firmicutes* (*Bacillota*) and *Tenericutes* (*Mycoplasmatota*), accompanied by decreased relative abundances of *Bacteroidetes* (*Bacteroidota*) and *Proteobacteria* (*Pseudomonadota*). JYH treatment partially reversed these RSV-induced alterations, suggesting a modulatory effect on the overall gut microbial structure (Figure 7A).

At the genus level, RSV infection increased the relative abundances of *Staphylococcus* and *Ruminococcus*, whereas the abundances of *Oscillospira* and *Bacteroides* were decreased. After JYH treatment, these changes were partially restored, suggesting that JYH modulated specific bacterial genera associated with RSV-induced gut microbiota dysbiosis (Figure 7B).

#### 3.3.3. Differential Gut Microbiota Analysis

**Figure 8:**
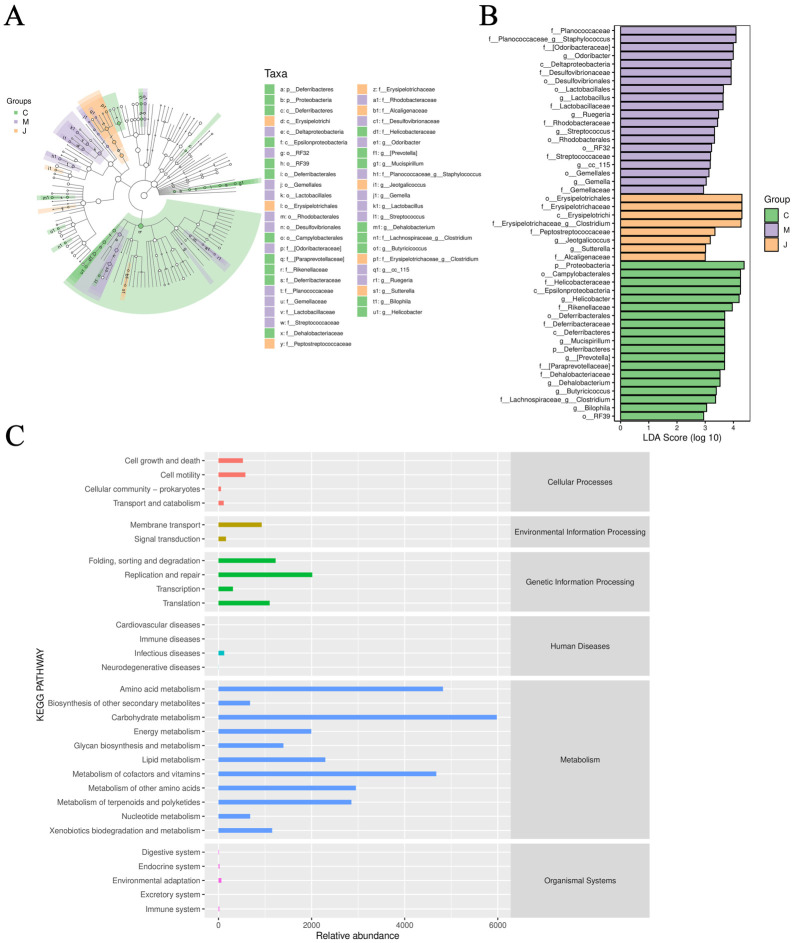
Differential gut microbiota and functional prediction analysis. (**A**) LEfSe analysis of differential bacterial taxa at the phylum level and the genus level. (**B**) Cladogram of LEfSe. (**C**) KEGG-based functional prediction of gut microbiota using PICRUSt2. C, control group; M, model group; J, JYH group. The LDA score threshold was set to >2.

To identify microbial taxa that differed significantly among groups, linear discriminant analysis effect size (LEfSe) was performed using a linear discriminant analysis (LDA) score threshold of >2. At the phylum level, *Deferribacteres* and *Firmicutes* were identified as key discriminative taxa in the model group compared with the control group, whereas *Proteobacteria* was the predominant discriminative taxon in the JYH group compared with the model group. At the genus level, *Ruegeria*, *Staphylococcus*, *Butyricicoccus*, and *Mucispirillum* were the main discriminative genera in the model group relative to the control group. In contrast, *Odoribacter*, *Lactobacillus*, and *Helicobacter* were identified as key discriminative genera in the JYH group compared with the model group (Figure 8A,B).

Functional prediction of the gut microbiota was further performed using PICRUSt2 based on 16S rRNA gene sequencing data, followed by annotation against the KEGG database. The predicted microbial functions were mainly enriched in metabolic pathways, particularly carbohydrate metabolism, amino acid metabolism, and cofactor and vitamin metabolism (Figure 8C). These results suggest that JYH may modulate not only the taxonomic composition of the gut microbiota but also its predicted metabolic functions in RSV-infected mice.

### 3.4. Correlation Analysis Between Differential Metabolites and Gut Microbiota

To investigate the potential associations between gut microbiota alterations and lung metabolic changes following JYH intervention, Spearman correlation analysis was conducted using the relative abundances of differential bacterial genera and the peak intensities of differential lung tissue metabolites.

**Figure 9:**
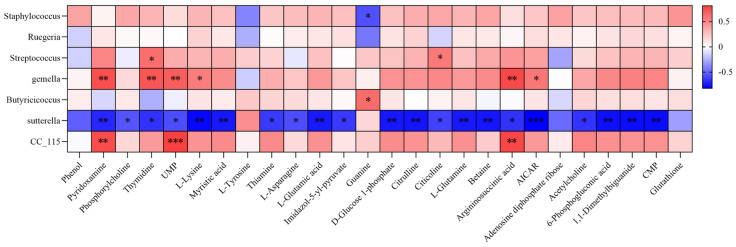
Heatmap of correlations between differentially expressed metabolites and gut microbiota. Heatmap showing Spearman correlations between differential lung tissue metabolites and differential gut microbial genera. *, ** and *** indicate *p* < 0.05, *p* < 0.01 and *p* < 0.001.

The correlation analysis suggested that JYH may be associated with coordinated alterations in lung tissue metabolism and gut microbiota composition during RSV infection. Several differential genera, including *Gemella*, *Sutterella*, and *CC_115*, showed significant correlations with multiple altered metabolites, including pyridoxamine, UMP, and argininosuccinic acid (Figure 9). These results indicate potential associations between JYH-related changes in the gut microbiota and pulmonary metabolic remodeling in RSV-infected mice.

## 4. Discussion

RSV infection has been reported to activate the Toll-like receptor 4/nuclear factor-kappa B (TLR4/NF-κB) signaling pathway, thereby promoting the transcription of inflammatory cytokines, including TNF-α, IL-6, and IL-8, and ultimately leading to excessive inflammatory responses and tissue damage [11]. In the present study, pharmacodynamic evaluation showed that JYH alleviated inflammatory cell infiltration and alveolar wall thickening, reduced RSV RNA levels in lung tissues, decreased IL-6 levels, and increased IFN-γ levels. These findings suggest that JYH attenuated RSV-induced pulmonary inflammation and pathological injury in mice.

Untargeted metabolomic analysis revealed that RSV infection induced extensive metabolic disturbances in lung tissues, with 46 differential metabolites identified between the control and model groups. Following JYH intervention, 26 differential metabolites tended to return toward normal levels. Pathway enrichment analysis showed that these metabolites were mainly associated with glutamatergic synapse, GABAergic synapse, arginine biosynthesis, glutathione metabolism, alanine, aspartate and glutamate metabolism, D-glutamine and D-glutamate metabolism, pyrimidine metabolism, and FoxO signaling. Although the nicotine addiction pathway was also enriched, its direct biological relevance to RSV-induced pulmonary infection remains unclear. This enrichment may reflect alterations in neurotransmitter-related metabolic networks rather than direct involvement of the nicotine addiction pathway in RSV pathogenesis.

Previous studies have shown that RSV infection can disrupt amino acid metabolism. For example, marked changes in amino acid profiles, including increased lysine, tyrosine, tryptophan, and phenylalanine levels and decreased arginine levels, have been observed in rats three days after RSV infection [12]. Other studies have identified GABA as a differential metabolite during RSV infection and highlighted D-glutamine and D-glutamate metabolism, as well as alanine, aspartate and glutamate metabolism, as important regulatory pathways [13,14]. Consistent with these findings, the present study showed that RSV infection disrupted glutamine and glutamate metabolism, whereas JYH intervention partially reversed these metabolic abnormalities. These results suggest that modulation of amino acid metabolism may be one of the mechanisms underlying the protective effects of JYH against RSV-induced pneumonia.

Glutathione, a tripeptide composed of glutamate, cysteine, and glycine, plays an important role in antioxidant defense and antiviral responses [15]. In this study, glutathione metabolism was altered in RSV-infected mice, which may be associated with viral infection-induced oxidative stress and immune activation. Following JYH intervention, glutathione metabolism tended to normalize, suggesting that JYH may contribute to maintaining redox balance and immune homeostasis during RSV infection. In addition, arginine biosynthesis was significantly enriched in the present study. A clinical follow-up study reported that recurrent wheezing in infants recovering from RSV bronchiolitis was associated with metabolites involved in the arginine biosynthesis pathway [16]. Therefore, modulation of arginine-related metabolism may also be involved in RSV-induced respiratory dysfunction and the therapeutic effects of JYH.

Glutamate and GABA are major excitatory and inhibitory neurotransmitters, respectively, and both are involved in regulating respiratory function [17,18]. RSV infection can cause cough, recurrent wheezing, and asthma-like complications, which may be associated with abnormal excitability of respiratory-related neuromuscular pathways [19,20]. In the present study, glutamate was identified as a significant differential metabolite in RSV-infected mice, and both glutamatergic synapse and GABAergic synapse pathways were markedly enriched. These findings suggest that RSV infection may affect neurotransmitter-related metabolic pathways in lung tissues, whereas JYH may help restore metabolic balance within these pathways. However, further experimental validation is required to clarify the functional relevance of these pathways in RSV-induced pulmonary injury.

Pyrimidine metabolism is also an important pathway involved in viral infection and antiviral responses. Previous studies have demonstrated that genes related to pyrimidine metabolism are highly expressed in human lung cells following SARS-CoV-2 infection [21]. Moreover, modulation of pyrimidine metabolism has been reported to contribute to the treatment of H1N1 influenza virus pneumonia [22]. In this study, KEGG enrichment analysis showed that pyrimidine metabolism was significantly altered in RSV-infected mice and regulated after JYH treatment. These findings suggest that JYH may influence nucleotide metabolism, which could be related to viral replication, host immune responses, and cellular repair processes during RSV infection.

Respiratory viral infections can also induce intestinal pathological injury and gut microbiota dysbiosis [23]. In this study, RSV infection altered the structure and composition of the gut microbiota, as evidenced by increased Firmicutes abundance and decreased Bacteroidetes and Proteobacteria abundance. Following JYH intervention, the relative abundances of Firmicutes and Bacteroidetes tended to return toward those observed in the control group. LEfSe analysis further revealed that RSV infection and JYH treatment were associated with changes in several characteristic bacterial genera, including *Gemella*, *Streptococcus*, *Staphylococcus*, *Butyricicoccus*, *Ruegeria*, *Sutterella*, and *CC_115*. These results suggest that JYH may ameliorate RSV-induced gut microbiota dysbiosis.

Several altered bacterial genera identified in this study have been associated with inflammation or respiratory diseases. *Gemella* is an opportunistic genus widely distributed on human mucosal surfaces. *Gemella haemolysans* has been reported to produce IgA1 protease at mucosal sites and has been associated with allergic diseases and meningitis [24,25]. *Staphylococcus* is a pyogenic bacterium that can cause infection, and its abundance has been reported to correlate with RSV infection severity [26]. *Streptococcus* has also been associated with pulmonary lesions and is frequently enriched in respiratory diseases such as pneumonia [27,28]. In addition, increased abundance of *CC_115* has been linked to intestinal inflammation [29]. In the present study, changes in these genera may reflect RSV-induced intestinal microecological disturbance and inflammatory responses.

In contrast, *Butyricicoccus* is generally considered a potentially beneficial genus that primarily colonizes the colonic mucosa and can synthesize butyrate from acetate [30,31]. Butyrate has been shown to exert multiple biological effects, including cardiovascular protection, attenuation of intestinal inflammation, promotion of anti-inflammatory factor secretion, inhibition of excessive pro-inflammatory cytokine release, and maintenance of intestinal mucosal barrier integrity [32,33,34,35]. In this study, *Butyricicoccus* abundance was markedly reduced in RSV-infected mice, which may contribute to impaired butyrate production, intestinal immune imbalance, and inflammatory dysregulation. JYH intervention increased the abundance of *Butyricicoccus*, suggesting that restoration of beneficial butyrate-producing bacteria may be involved in its protective effects.

*Sutterella*, a dominant genus within the phylum Proteobacteria, has been reported to decrease in the gut microbiota of patients with asthma and to increase after effective intervention [36]. In the present study, *Sutterella* abundance was lower in RSV-infected model mice than in control mice, whereas JYH treatment increased its abundance. This result suggests that *Sutterella* may be associated with the regulatory effects of JYH on gut microbiota dysbiosis in RSV-infected mice. However, because the functional role of *Sutterella* can vary across disease contexts, further studies are required to determine its specific contribution to RSV-associated pulmonary inflammation.

The gut microbiota plays a critical role in pulmonary immunity and host defense against respiratory viral infections. To further explore the relationship between pulmonary metabolic remodeling and intestinal microecological changes, Spearman correlation analysis was performed between differential metabolites and differential gut microbiota. The results showed significant correlations between several key metabolites and characteristic bacterial genera, suggesting a potential association between host metabolic disturbance and gut microbiota dysbiosis. These findings provide multi-omics evidence supporting the potential involvement of the gut–lung axis in the protective effects of JYH against RSV-induced pneumonia. Nevertheless, correlation analysis does not establish causality, and further mechanistic studies are needed to determine whether specific microbial taxa directly regulate pulmonary metabolites or inflammatory responses.

Overall, this study systematically investigated the protective effects of JYH against RSV-induced pneumonia using pharmacodynamic evaluation, untargeted metabolomics, and gut microbiota profiling. However, several limitations should be acknowledged. First, the signaling pathways and microbial functions identified in this study were inferred from untargeted metabolomic enrichment analysis and 16S rRNA-based functional prediction; targeted molecular validation, pathway intervention experiments, and microbial metabolite measurements were not performed. Second, this study revealed associations between differential metabolites and gut microbiota, but the causal relationships and specific regulatory mechanisms remain unclear. Therefore, future studies involving targeted pathway validation, fecal microbiota transplantation, and characteristic metabolite intervention are warranted to further clarify the mechanisms by which JYH modulates gut microbiota and metabolic homeostasis in RSV-induced pneumonia.

## 5. Conclusions

In this study, the therapeutic effects and potential mechanisms of JYH against RSV-induced pneumonia were investigated using an RSV-infected mouse model. JYH alleviated pulmonary pathological injury, reduced viral load, decreased IL-6 levels, and increased IFN-γ levels in RSV-infected mice. Integrated metabolomic and gut microbiota analyses further suggested that JYH may exert protective effects by modulating pulmonary metabolic disturbances and gut microbiota dysbiosis. These findings provide new insights into the potential mechanisms underlying the anti-RSV effects of JYH. However, the proposed interactions between metabolites and the gut microbiota remain predictive and associative; therefore, further experimental validation is required to confirm the underlying mechanisms of action.

## Figures and Tables

**Table 1:** Primer sequences used for qRT-PCR detection in the RSV-infected mouse model.

Name	Primer Sequences
RSV-N	F: 5′-ATA CAC CAT CCA ACG GAG CAC-3′
	R: 5′-GTG TCT TCT CTT CCT AAC CTA-3′
PPIA	F: 5′-CGC TTG CTG CAG CCA TGG TC-3′
	R: 5′-CAG CTC GAA GGA GAC GCG GC-3′

## Data Availability

Data will be made available on request.

## Supplementary Materials

The following supporting information can be downloaded at: https://www.mdpi.com/article/10.3390/metabo16060360/s1. Figure S1: Results of quality assurance. (**A**) Positive ion mode. (**B**) Negative ion mode.; Figure S2: The Regulatory Role of JYH in Differential Metabolites. C, control group; M, model group; J, JYH group. *, ** and *** indicate *p* < 0.05, *p* < 0.01 and *p* < 0.001.

## References

[1] Meng X., Wang S.C., Xie T., Xu J.Y., Shen C.S., Shan J.J. (2017). Biomarkers in Urine and Feces of BALB/c Mice Infected with RSV Pneumonia Using GC-MS Technology. J. Nanjing Univ. Tradit. Chin. Med..

[2] Wu H., Liu Z., Li Y. (2025). Intestinal microbiota and respiratory system diseases: Relationships with three common respiratory virus infections. Microb. Pathog..

[3] Zheng H.T., Zhao Q.Y., Ding Y., Ma S.X., Chen W.X., Qiu J.L., Li X.F., Sun X.X., Zhang Y.J., Yuan B. (2023). Investigation of the relationships among respiratory syncytial virus infection, T cell immune response and intestinal flora. Eur. Rev. Med. Pharmacol. Sci..

[4] Shan J., Qian W., Shen C., Lin L., Xie T., Peng L., Xu J., Yang R., Ji J., Zhao X. (2018). High-resolution lipidomics reveals dysregulation of lipid metabolism in respiratory syncytial virus pneumonia mice. RSC Adv..

[5] Teoh S.T., Leimanis-Laurens M.L., Comstock S.S., Winters J.W., Vandenbosch N.L., Prokop J.W., Bachmann A.S., Lunt S.Y., Rajasekaran S. (2022). Combined Plasma and Urinary Metabolomics Uncover Metabolic Perturbations Associated with Severe Respiratory Syncytial Viral Infection and Future Development of Asthma in Infant Patients. Metabolites.

[6] Li W., Zhang L., He P., Li H., Pan X., Zhang W., Xiao M., He F. (2024). Traditional uses, botany, phytochemistry, and pharmacology of Lonicerae japonicae flos and Lonicerae flos: A systematic comparative review. J. Ethnopharmacol..

[7] Zheng T.Y., Liu W.J., Dong S.Q., Shao S.J., Feng J.L., Yang F.D. (2025). Research progress on the extraction, composition analysis and pharmacological effects of volatile oil of Lonicera japonica. West China J. Pharm. Sci..

[8] Liang Y., Liu M., Wang Y., Liu L., Gao Y. (2023). Analyzing the Material Basis of Anti-RSV Efficacy of Lonicerae japonicae Flos Based on the PK-PD Model. Molecules.

[9] Ding J., Yan G.L., Yang P., Zhang Y.Q., Liu Y.H. (2020). Study on Fingerprint and in vitro Antiviral Activity of Lonicera japonica Polysaccharide. China Pharm..

[10] Ding Y., Cao Z., Cao L., Ding G., Wang Z., Xiao W. (2017). Antiviral activity of chlorogenic acid against influenza A (H1N1/H3N2) virus and its inhibition of neuraminidase. Sci. Rep..

[11] Li C., Lv J., Yang L.F., Zhao B.N., Gao Y. (2021). Integrate network pharmacology to explore the anti-RSV pneumonia mechanism of Lonicera Japonica Thunb. based upon UPLC-Q-Exactive-Orbitrap-MS. Chin. J. Hosp. Pharm..

[12] Ouyang Y., Chi L., Xu C., Zhao X.J., Cui Z.Z. (2021). Liquid chromatography-mass spectrometry-based metabolomics study of the efficacy of Chinese medicine asthma-relieving decoction on respiratory syncytial virus infection. Chin. J. Chromatogr..

[13] Meng X., Shan J.J., Xie T., Xu J.Y., Shen C.S., Wang S.C. (2016). Antiviral Effects of Jinxin Oral Liquid Against Respiratory Syncytial Virus Infection in Vitro Based on Gas Chromatography-Mass Spectrometer Metabolomics. Liaoning J. Tradit. Chin. Med..

[14] Meng X., Wang S.C., Shan J.J., Xu J.Y., Shen C.S., Xie T. (2016). Regulation of Jinxin Oral Liquid on metabolites in spleen of mice with RSV pneumonia based on GC-MS. Chin. Tradit. Herb. Drugs.

[15] Wang X., Dong Q.-H. (2021). Progress in the study of the inhibitory effect of glutathione on viruses and its interaction with molecules in vivo. Health Med. Res. Pract..

[16] Zhang X., Peng D., Zhang X., Wang X., Chen N., Zhao S., He Q. (2021). Serum metabolomic profiling reveals important difference between infants with and without subsequent recurrent wheezing in later childhood after RSV bronchiolitis. APMIS Acta Pathol. Microbiol. Immunol. Scand..

[17] Cherubini E., Conti F. (2001). Generating diversity at GABAergic synapses. Trends Neurosci..

[18] Conti F., Weinberg R.J. (1999). Weinberg, Shaping excitation at glutamatergic synapses. Trends Neurosci..

[19] Li D.Y., Zhao Y.D. (2021). Clinical characteristics and serum cytokine profile of children with acute exacerbation of bronchial asthma induced by respiratory syncytial virus infection. Clin. Educ. Gen. Pract..

[20] Sheng M.L., Shao Q.M., Zhang C.L. (2021). Analysis of influencing factors of recurrent wheezing in children with respiratory syncytial virus bronchiolitis. Matern. Child Health Care China.

[21] Yang S., Wu S., Yu Z., Huang J., Zhong X., Liu X., Zhu H., Xiao L., Deng Q., Sun W. (2020). Transcriptomic analysis reveals novel mechanisms of SARS-CoV-2 infection in human lung cells. Immun. Inflamm. Dis..

[22] Qian W.J., Yang R., Xie T., Yao W.F., Kang A., Di L.Q., Shan J.J. (2018). Metabolomics on Pudilan Xiaoyan Oral Liquid in treatment of Influenza A/H1N1-induced pneumonia based on GC-MS. Chin. Tradit. Herb. Drugs.

[23] Willing B.P., Dicksved J., Halfvarson J., Andersson A.F., Lucio M., Zheng Z., Järnerot G., Tysk C., Jansson J.-K., Engstrand L. (2010). A pyrosequencing study in twins shows that gastrointestinal microbial profiles vary with inflammatory bowel disease phenotypes. Gastroenterology.

[24] García López E., Martín-Galiano A.J. (2020). The Versatility of Opportunistic Infections Caused by Gemella Isolates Is Supported by the Carriage of Virulence Factors From Multiple Origins. Front. Microbiol..

[25] Dzidic M., Abrahamsson T.R., Artacho A., Collado M.C., Mira A., Jenmalm M.C. (2018). Oral microbiota maturation during the first 7 years of life in relation to allergy development. Allergy.

[26] Sonawane A.R., Tian L., Chu C.Y., Qiu X., Wang L., Holden-Wiltse J., Grier A., Gill S.R., Caserta M.T., Falsey A.R. (2019). Microbiome-Transcriptome Interactions Related to Severity of Respiratory Syncytial Virus Infection. Sci. Rep..

[27] Gutbier B., Fischer K., Doehn J.M., von Lachner C., Herr C., Klaile E., Frischmann U., Singer B.-B., Riesbeck K., Zimmermann W. (2015). Moraxella catarrhalis induces an immune response in the murine lung that is independent of human CEACAM5 expression and long-term smoke exposure. Am. J. Physiol.-Lung Cell. Mol. Physiol..

[28] Riise G.C., Qvarfordt I., Larsson S. (2000). Inhibitory effect of N-acetylcysteine on adherence of Streptococcus pneumoniae and Haemophilus influenzae to human oropharyngeal epithelial cells in vitro. Respiration.

[29] Latorre J.D., Adhikari B., Park S.H., Teague K.D., Graham L.E., Mahaffey B.D., Baxter M.F.A., Hernandez-Velasco X., Kwon Y.M., Ricke S.C. (2018). Evaluation of the Epithelial Barrier Function and Ileal Microbiome in an Established Necrotic Enteritis Challenge Model in Broiler Chickens. Front. Vet. Sci..

[30] Chen J., Bittinger K., Charlson E.S., Hoffmann C., Lewis J., Wu G.D., Collman R.G., Bushman F.D., Li H. (2012). Associating microbiome composition with environmental covariates using generalized UniFrac distances. Bioinformatics.

[31] Geirnaert A., Steyaert A., Eeckhaut V., Debruyne B., Arends J.B., Van Immerseel F., Boon N., Van de Wiele T. (2014). Butyricicoccus pullicaecorum, a butyrate producer with probiotic potential, is intrinsically tolerant to stomach and small intestine conditions. Anaerobe.

[32] Steppe M., Van Nieuwerburgh F., Vercauteren G., Boyen F., Eeckhaut V., Deforce D., Haesebrouck F., Ducatelle R., Van Immerseel F. (2014). Safety assessment of the butyrate-producing Butyricicoccus pullicaecorum strain 25-3(T), a potential probiotic for patients with inflammatory bowel disease, based on oral toxicity tests and whole genome sequencing. Food Chem. Toxicol. Int. J. Publ. Br. Ind. Biol. Res. Assoc..

[33] Eeckhaut V., Machiels K., Perrier C., Romero C., Maes S., Flahou B., Steppe M., Haesebrouck F., Sas B., Ducatelle R. (2013). Butyricicoccus pullicaecorum in inflammatory bowel disease. Gut.

[34] Eeckhaut V., Wang J., Van Parys A., Haesebrouck F., Joossens M., Falony G., Raes J., Ducatelle R., Van Immerseel F. (2016). The Probiotic Butyricicoccus pullicaecorum Reduces Feed Conversion and Protects from Potentially Harmful Intestinal Microorganisms and Necrotic Enteritis in Broilers. Front. Microbiol..

[35] Zhou Z.Q., Jiang Q., Tang X.P., Zhang J.Y., Li K.S., Peng Q.W., Wang J. (2020). Characteristics of intestinal flora in patients with primary Sjögren’s syndrome with deficiency of both qi and yin. China J. Tradit. Chin. Med. Pharm..

[36] Huang C., Yu Y., Du W., Liu Y., Dai R., Wang P., Zhang C., Shi G. (2021). Insights into gut microbiome and its functional pathways in asthma patients through high-throughput sequencing. Future Microbiol..

