# Supplementary material for: Integrating Metabolomics and Gut Microbiota to Reveal the Therapeutic Effect of Lonicerae japonicae Flos Against Respiratory Syncytial Virus
Source: Metabolites. 2026 May 27;16(6):360. doi: 10.3390/metabo16060360 (PMC13303406; doi:10.3390/metabo16060360)
Supplement: Supplementary file 1 [file metabolites-16-00360-s001.zip › metabolites-4288023-supplementary.pdf]

Supplementary Materials

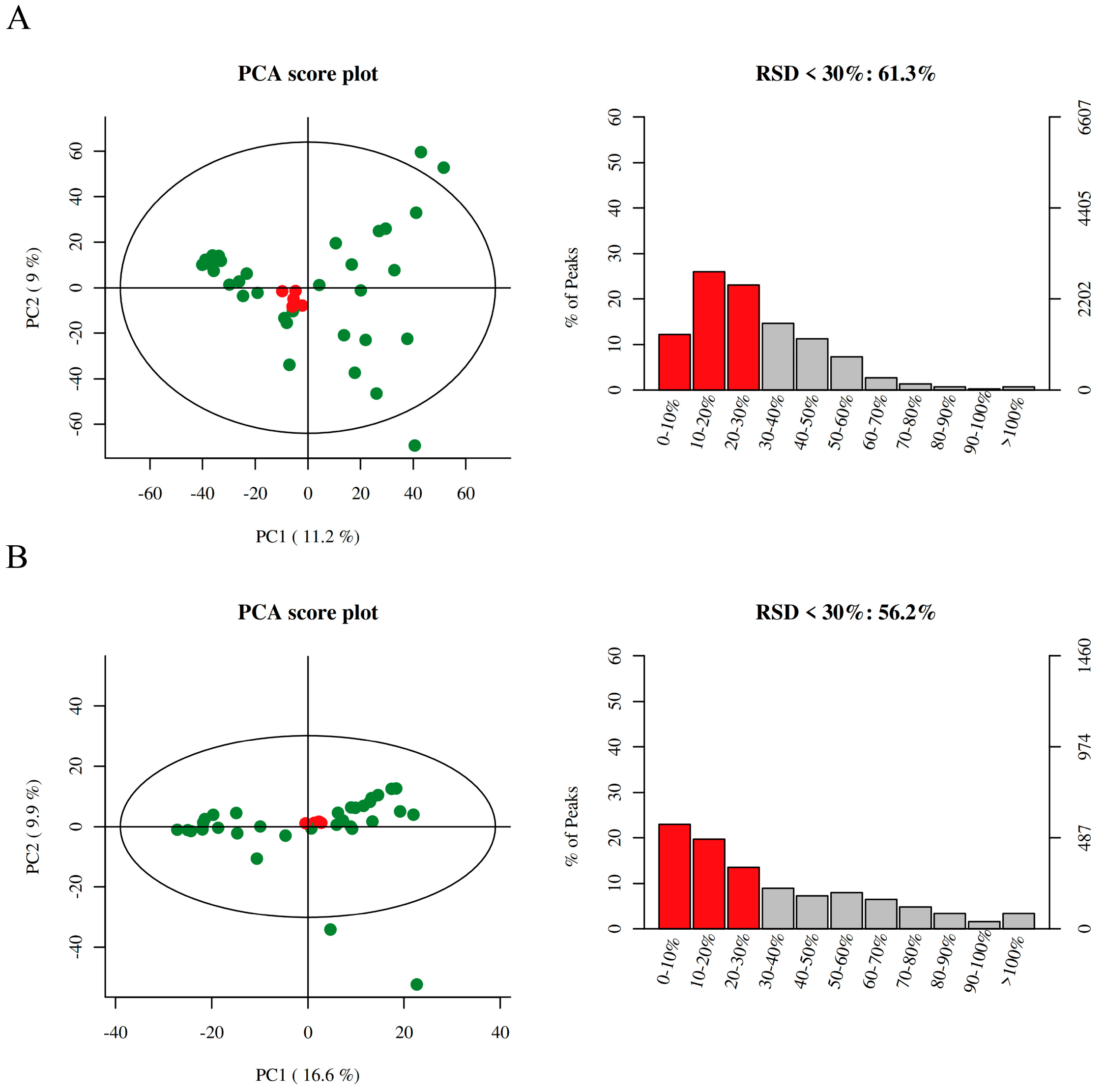

Figures S1. Results of quality assurance. (A) Positive ion mode. (B) Negative ion mode.

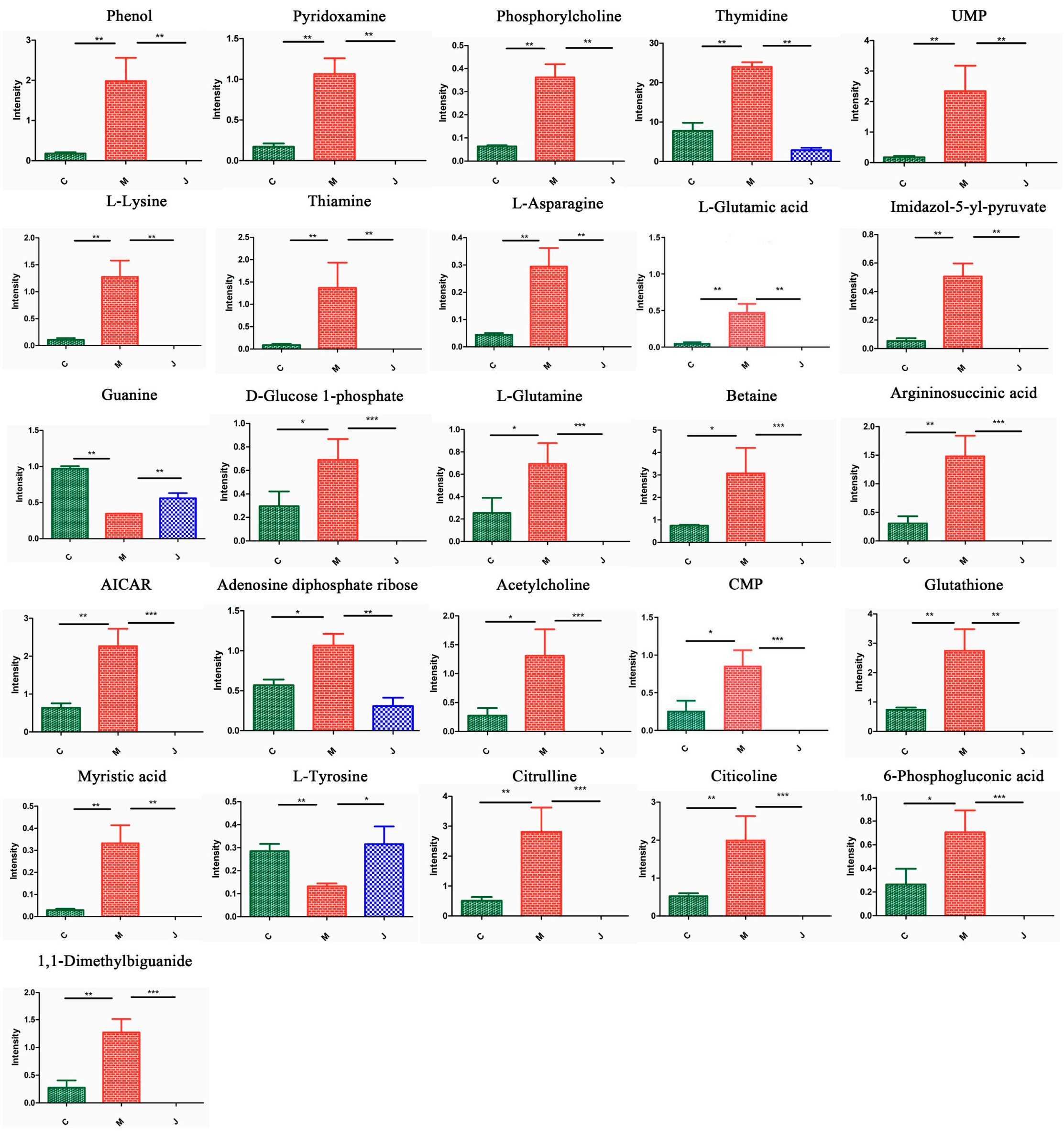

Figures S2. The Regulatory Role of JYH in Differential Metabolites. C, control group; M, model group; J, JYH group. *, ** and *** indicate $P < 0.05$, $P < 0.01$ and $P < 0.001$.
